# Disease specific urinary biomarkers in the central nervous system

**DOI:** 10.1038/s41598-023-46763-z

**Published:** 2023-11-07

**Authors:** Micah Duggins-Warf, Aram Ghalali, Julie Sesen, Tyra Martinez, Katie P. Fehnel, Steven Pineda, David Zurakowski, Edward R. Smith

**Affiliations:** 1 Vascular Biology Program, Boston Children’s Hospital, 300 Longwood Avenue, Boston, MA 02115 USA; 2 Department of Neurosurgery, Boston Children’s Hospital, Boston, MA USA; 3 Department of Surgery, Boston Children’s Hospital, Boston, MA USA

**Keywords:** Cancer, Molecular biology, Neuroscience, Biomarkers, Cardiology, Diseases, Medical research, Molecular medicine, Neurology, Oncology, Pathogenesis

## Abstract

Urinary biomarkers can diagnose and monitor pathophysiologic conditions in the central nervous system (CNS). However, focus is often on single diseases, with limited data on discriminatory capability of this approach in a general setting. Here, we demonstrate that different classes of CNS disease exhibit distinct biomarker patterns, evidence of disease-specific “fingerprinting.” Urine from 218 patients with pathology-confirmed tumors or cerebrovascular disease, controls (n = 33) were collected. ELISA and/or bead-based multiplexing quantified levels of 21 putative urinary biomarkers. Analysis identified biomarkers capable of distinguishing each disease from controls and other diseases. Mann–Whitney U tests identified biomarkers with differential expression between disease types and controls (P ≤ 0.001). Subsequent receiver-operating characteristic (ROC) analyses revealed distinguishing biomarkers with high sensitivity and specificity. Areas under the curve (AUCs) ranged 0.8563–1.000 (P values ≤ 0.0003), sensitivities ranged 80.00–100.00%, and specificities ranged 80.95–100.00%. These data demonstrate proof-of-principle evidence that disease-specific urinary biomarker signatures exist. In contrast to non-specific responses to ischemia or injury, these results suggest that urinary biomarkers accurately reflect unique biological processes distinct to different diseases. This work can be used to generate disease-specific panels for enhancing diagnosis, assisting less-invasive follow-up and herald utility by revealing putative disease-specific therapeutic targets.

## Introduction

Despite advances in imaging techniques, the ability to identify, diagnose and provide easy, reliable follow-up of many neurosurgical diseases, particularly high-risk brain tumors and stroke conditions, remains challenging. Consequently, there has been a strong impetus to promote the investigation and development of novel diagnostic and prognostic methodologies to enhance the capabilities for clinicians treating patients with these diseases. Biomarkers have emerged as increasingly important and useful tools to complement neuroimaging^1,2^. Our laboratory has explored the utility of urinary biomarkers in the management of these diseases^1–6^.

**Figure 1:**
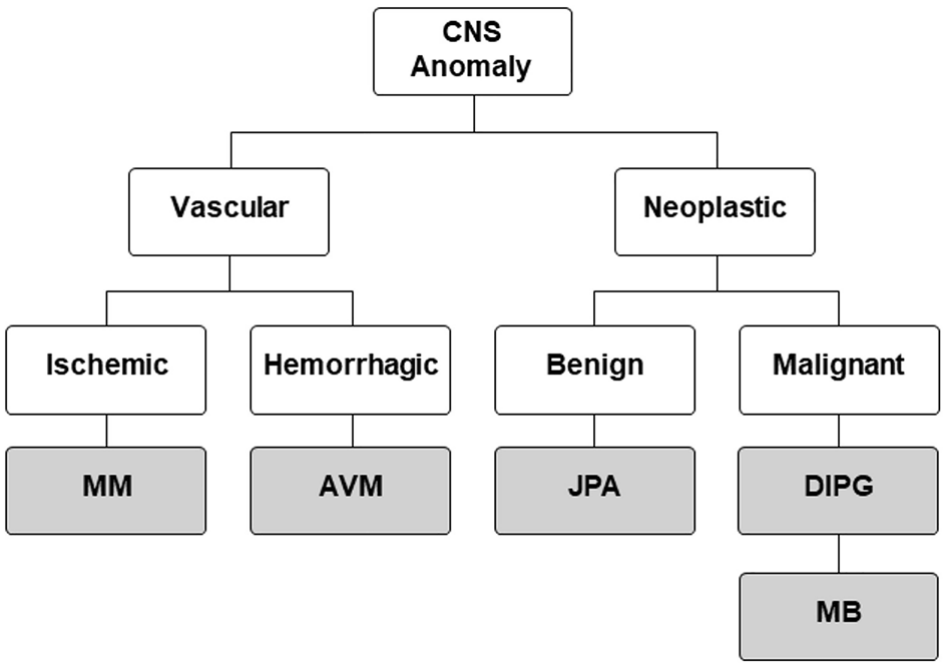
Disease tree. *CNS* central nervous system, *MM* MoyaMoya, *AVM* arterio-veinous malformation, *JPA* Juvenile pilocytic astrocytoma, *DIPG* diffuse intrinsic pontine glioma, *MB* medulloblastoma.

In this study, we provide proof-of-principle data that urinary biomarkers can discriminate between multiple central nervous system (CNS) diseases (Fig. 1), including being able to distinguish subtypes of disease, such as unique tumor pathologies or stroke conditions. The work described here builds on our previously published reports of tissue-validated and longitudinal studies of urinary biomarkers in these disorders^1–6^. In combination with the new data presented here, we establish a foundation for future studies predicated on leveraging this unique, cheap and non-invasive technique to aid clinical care.

## Methods

### Patient population

Given the large clinical impact of tumor and stroke/cerebrovascular disease, representative diseases from these groups were selected for study (Table 1). The cerebrovascular conditions included moyamoya disease (MM) and arteriovenous malformations (AVM). The tumor conditions included three neoplastic lesions, including one benign lesion, juvenile pilocytic astrocytoma (JPA) and two malignant lesions, diffuse intrinsic pontine glioma (DIPG) and medulloblastoma (MB). All disease diagnoses were confirmed as part of routine clinical practice, with validation by imaging review with neuroradiology and neuropathology confirming tissue diagnosis (for all tumors and AVMs).

**Table 1:** Number, average age and sex of patients with the selected five CNS abnormalities and those included as control in the study.

Diagnosis	Number of patients	Age: avg. ± SD	Sex: M (F)
Control	33	5.70 ± 3.82	12 (21)
MM	51	8.98 ± 5.68	16 (35)
AVM	34	11.44 ± 4.18	16 (18)
JPA	41	9.24 ± 5.35	22 (19)
DIPG	30	7.37 ± 3.03	14 (16)
MB	29	8.45 ± 4.69	14 (15)

The five pathologies are moyamoya disease (MM), arteriovenous malformations (AVM), juvenile pilocytic astrocytoma (JPA), diffuse intrinsic pontine glioma (DIPG) and medulloblastoma (MB).

All samples and data were collected and analyzed under institutional review board (IRB) approval from Boston Children’s Hospital and all methods were performed in accordance with the relevant guidelines and regulations. Furthermore, informed consent was obtained from all subjects and/or their legal guardian(s).

A total of 218 urine samples (Table 1) were analyzed from patients with different CNS anomalies including a group of patients with fatty filum (FF) used as control for the study to ensure that our diagnostic panels could not only differentiate between diseases but were also distinct from healthy control subjects. Our group and others have previously validated FF patients as a control cohort for biomarker studies, given the advantageous combination of FF being a congenital finding, coupled with those individuals also having neuroimaging to exclude other pathologies that might confound analysis^7^.

### Urinary biomarker panel

Our panel of biomarkers consisted of 21 proteins known to be excreted through micturition. These biomarkers are matrix metalloproteinases (MMP-2, MMP-3, MMP-9, MMP-13), growth factors (EGF, bFGF, HB-EGF, HGF, PlGF, VEGF), cytokines (Angiogenin, Angiopoietin-1, Netrin-1, Prosaposin, Thrombospondin-1, Thrombospondin-2), cysteine proteases (Cathepsin B), or tissue inhibitors of metalloproteinases (TIMP-1, TIMP-2, TIMP-3, TIMP-4).

### Urine collection

Urine from 218 patients (0–25 years of age, the youngest patient was 6 months of age), with medulloblastoma (n = 29), juvenile pilocytic astrocytoma (n = 41), moyamoya syndrome (n = 51), arteriovenous malformation (n = 34), or diffuse intrinsic pontine glioma (n = 30), along with controls (n = 33) was collected under an IRB approved protocol and all methods were performed in accordance with the relevant guidelines and regulations. Furthermore, informed consent was obtained from all subjects and/or their legal guardian(s).

All tumor and AVM samples were confirmed with pathologic analysis performed as part of routine clinical care by neuropathologists. Moyamoya does not result in pathology to be analyzed; the diagnosis is made by radiographic and clinical criteria^8,9^. Urine was collected via catheter prior to index or any surgical, chemotherapeutic, or radiotherapeutic intervention for their disease. Specimens were placed on ice and collected from the operating room < 3 h by laboratory personnel. Upon collection, specimens were vortexed to homogenize the content, aliquoted into microcentrifuge tubes, and stored at − 80 °C. ELISA and/or bead-based multiplexing were performed on our samples to quantify the levels of an established panel of 21 putative urinary biomarkers, and these values were normalized to total protein concentration using Bradford assays. These values were then statistically analyzed to look for biomarkers that would distinguish each disease both from controls, and from the other diseases.

### Protein analysis

The panel of proteins selected for analysis was derived from previous work in our lab and others confirming that several of these molecules were present in each of the selected pathologies, were able to be detected in the urine and had commercially available assays to allow for validation^2–6^. All samples were assayed for total protein concentration by mixing 100 μL of urine with 900 μL of a solution of 2:7 Bradford dye (Bio-Rad Laboratories, Inc.) and distilled water. Spectral absorbance of these samples at 595 nm was determined using a Beckman DU 640 spectrophotometer, and protein concentration was determined using Bovine Serum Albumin (BSA) standard curve.

Total Netrin-1 levels were determined by ELISA (US Biomax, Inc.) as were Thrombospondin-1 and Cathepsin-B levels (R&D Systems, Inc.). ELISAs were performed according to manufacturer instructions, and were read using a FilterMax F3 spectrophotometer (Molecular Devices). Levels of MMP-2, MMP-3, MMP-9, MMP-13, Angiogenin, Angiopoietin, PIGF, Thrombospondin-2, VEGF, EGF, HGF, HB-EGF and FGFb were analyzed using custom Luminex® screening assays (R&D Systems, Inc.). TIMP-1, TIMP-2, TIMP-3, and TIMP-4 levels were analyzed using a Luminex® performance assay (R&D Systems, Inc.). All Luminex® assays were performed according to manufacturer instructions, and read using a Bio-Plex 200 HTF system (Bio-Rad Laboratories, Inc.).

Protein concentrations are given in picograms per microgram (pg/μg), and were determined by dividing the concentration of the target protein in the sample (pg/mL) by the concentration of total protein in the sample (μg/mL) as a normalization measure.

### Statistical analysis

All statistical analysis was carried out by a dedicated biostatistician (DZ).

Grubb’s Test for Outliers^10^ with sensitivity α = 0.05 was applied iteratively to each data set to correct for experimental error. Given that much of our data did not follow a normal distribution, nonparametric Mann–Whitney U tests were then used to compare the levels of a given analyte in one disease population to the levels of that analyte in every other disease population in the group, and the control population.

The proteins that showed significant potential (P ≤ 0.001) in differentiating one disease from another disease or control were subjected to ROC (receiver operating characteristic) analysis. The best protein for each comparison was then selected based on the AUC (area under the curve) for each ROC analysis. AUC values for the best biomarkers were gathered, along with the P value of the AUC. A protein cutoff level for a putative diagnostic test using these results was determined as the point on the ROC curve where the sum of sensitivity and specificity was closest to 200%.

All statistical analyses were performed using Prism 6 for Macintosh OS X (GraphPad Software, Inc.).

This biomarker study is presented in accordance with REporting recommendations for tumor MARKer prognostic studies (REMARK criteria)^11^.

## Results

### Urinary biomarkers are capable of distinguishing individual disease cohorts

By applying the Grubbs Test iteratively, a total of 337 extreme values were removed from a total of 4140 data points (12.29%). This allowed us to account for variation inherent to these assays. Mann–Whitney U tests of this trimmed data revealed markers that were capable of distinguishing between diseases, or between disease and control (Table 2). The untrimmed heat map is presented in supplemental data (Supplemental Table S1) and includes a number of additional putative biomarker candidates that were part of the initial analyses, but not found to be as significant as the species presented in Table 2.

**Table 2:** Heat map showing the proteins which are different comparing two diseases or a disease to control.

	Ctrl vs. JPA	Ctrl vs. MB	Ctrl vs. MM	Ctrl vs. AVM	Ctrl vs. DIPG	JPA vs. MB	JPA vs. MM	JPA vs. AVM	JPA vs. DIPG	MB vs. MM	MB vs. AVM	MB vs. DIPG	MM vs. AVM	MM vs. DIPG	AVM vs. DIPG
Angiopoietin -1		*	**			**	*			****		*	**	*	
PIGF			*		*		*		*						
MMP-2															
MMP-3		**	****	*	***		***		*	**			**		
MMP-9			**			*	****	*	**						
MMP-13	*	**	***			****	****				*	*	****	***	
TIMP-1	****	*		*	****		****			**			**	****	
TIMP-2	*														
TIMP-3							**	*						*	*
TIMP-4	****	***		***	***		**		****	*		****	*	****	****
HGF															
VEGF			*	**				*						**	**
Netrin-1					*	*			***		*			**	***
EGF	*	***	****	****	*										
bFGF	***		***		**			*					*		*
Angiogenin				*											
Angiopoietin-2			**	*						*				*	
Thrombospondin-1	*	*	****	*	*		***			*		**	*		
Thrombospondin-2			****		*		**			**			*		
HB-EGF			****	*	**		****	**	***	***	**	***	****	****	*

*p < 0.05, **p < 0.01, ***p < 0.001, and ****p < 0.0001. However, considering the choice of an alpha significance level of < 0.001 due multiple testing indicating that only *** and **** are convincing enough to be deemed as statistically significant.

Subsequent individual cohort analysis revealed discriminatory biomarkers for each disease. As an example, when comparing MB to DIPG, Angiopoietin-1, MMP-13, TIMP-4, Thrombospondin-1 and HB-EGF were all found to be useful as differentiating markers. TIMP-4, however, showed the most significant difference in expression between these two populations. The same was true when DIPG was compared to JPA. The other distinguishing markers for DIPG were TIMP-1 (vs. Ctrl and MM), HB-EGF (vs. JPA, MB and MM), MMP-3 (vs. Ctrl) and Netrin-1 (vs. JPA and AVM). These five biomarkers, along with their cutoff points, we consider to be the biomarker “fingerprint” for DIPG.

Using this methodology, a unique fingerprint was generated for each population in our study, comprised of a specific protein biomarker and cutoff level. Subsequent receiver-operating characteristic (ROC) analysis of the most statistically significant biomarker for each disease-disease or disease-control comparison produced area under the curve (AUC), sensitivity, and specificity figures for each cohort. The average AUC was 0.8121 ± 0.0593, and the average sensitivity and specificity were 82.15% ± 11.67% and 77.03% ± 17.66%, respectively (Table 3). The biomarker with the highest AUC (0.89) is MMP-3 to distinguish the control patients from patients with MM.

**Table 3:** Univariate analyses showing the ability of urinary biomarkers to discriminate between two cerebrovascular diseases or between a disease and the control.

Comparison	Disease	Versus	Dx #	Vs. #	Analyte	AUROCC	P value	C/o (pg/ug)	Sensitivity (%)	Specificity (%)
1	JPA	DIPG	28	28	TIMP-4	0.8750	< 0.0001	> 0.01897	75.00	100.00
2	JPA	MM	37	49	MMP-13	0.7882	< 0.0001	> 0.4168	73.47	75.68
3	JPA	Ctrl	29	39	TIMP-1	0.7790	< 0.0001	> 6.446	69.23	75.86
4	JPA	MB	37	27	MMP-13	0.7668	0.0003	> 0.7555	74.07	78.38
5	JPA	AVM	38	19	HB-EGF	0.7105	0.0101	< 0.0023	100.00	42.11
6	MB	DIPG	19	28	TIMP-4	0.8750	< 0.0001	> 0.01897	75.00	100.00
7	MB	Ctrl	23	25	EGF	0.7791	0.0009	> 265.2	72.00	73.91
8	MB	MM	27	43	Angiopoietin-1	0.7786	< 0.0001	> 0.4163	76.74	70.37
9	MB	JPA	37	27	MMP-13	0.7668	0.0003	> 0.7555	74.07	78.38
10	MB	AVM	21	19	HB-EGF	0.7143	0.0206	< 0.03463	100.00	42.86
11	MM	Ctrl	26	50	MMP-3	0.8900	< 0.0001	> 0.1968	84.00	84.62
12	MM	AVM	33	19	HB-EGF	0.8636	< 0.0001	< 0.03913	100.00	72.73
13	MM	DIPG	33	23	HB-EGF	0.8636	< 0.0001	< 0.03913	100.00	72.73
14	MM	JPA	37	49	MMP-13	0.7882	< 0.0001	> 0.4168	73.47	75.68
15	MM	MB	27	43	Angiopoietin-1	0.7786	< 0.0001	> 0.4163	76.74	70.37
16	AVM	DIPG	23	28	TIMP-4	0.8750	< 0.0001	> 0.01897	75.00	100.00
17	AVM	MM	33	19	HB-EGF	0.8636	< 0.0001	< 0.03913	100.00	72.73
18	AVM	Ctrl	23	27	EGF	0.8213	0.0001	> 215.2	81.48	73.91
19	AVM	MB	21	19	HB-EGF	0.7143	0.0206	< 0.03463	100.00	42.86
20	AVM	JPA	38	19	HB-EGF	0.7105	0.0101	< 0.0023	100.00	42.11
21	DIPG	AVM	23	28	TIMP-4	0.8750	< 0.0001	> 0.01897	75.00	100.00
22	DIPG	JPA	28	28	TIMP-4	0.8750	< 0.0001	> 0.01897	75.00	100.00
23	DIPG	MB	19	28	TIMP-4	0.8750	< 0.0001	> 0.01897	75.00	100.00
24	DIPG	MM	33	23	HB-EGF	0.8636	< 0.0001	< 0.03913	100.00	72.73
25	DIPG	Ctrl	26	21	MMP-3	0.8022	0.0004	> 0.3214	76.19	92.31

AUROCC analyses, the optimal cut-off (C/O), the sensitivity and the specificity are given for the most relevant comparisons. *AUROCC* area under receiver-operating characteristic curve, *JPA* Juvenile pilocytic astrocytoma, *MB* medulloblastoma, *MM* moyamoya syndrome, *AVM* arteriovenous malformation, *DIPG* diffuse intrinsic pontine glioma.

### Urinary biomarker analysis reveals distinct “fingerprints” by which populations can be identified

As Table 3 shows, for every disease-disease or disease-control comparison, each cohort had a specific protein biomarker with significant discriminatory capability. These fingerprints are summated in a diagnostic map that allows for immediate comparisons between diseases with their cut-off point—a “heat map” of putative biomarkers (Table 4). The protein cutoff level was determined as the point on the ROC curve where the sum of sensitivity and specificity was closest to 200%. The supplemental data S1 shows another potential representation to highlight specific fingerprint. The analytes with their specific cut-off level are presented in supp. Fig. S1A and the supp. Fig. S1B represents the distribution of the protein expression for the control. The distinct protein expressions are illustrated in supp. Fig. S1 with the specific fingerprints for the studied diseases. For the purpose of attempting to characterize the diseases in this study by speed of progression and rapidity of symptom onset, we propose this rank list, sorting the conditions from those with longest duration of disease presentation (JPAs may take years to grow into symptomatic lesions, versus DIPG may take weeks to months, while MM can cause ischemia in days).

**Table 4:** Table illustrating a possible diagnostic map for clinicians.

Ctrl	JPA	MB	MM	AVM	DIPG	Vs
	< 6.466 pg/μg TIMP-1 AUC: 0.7790	< 265.2 pg/μg EGF AUC: 0.7791	< 0.197 pg/μg MMP-3 AUC: 0.8900	< 215.2 pg/μg EGF AUC: 0.8213	< 0.321 pg/μg MMP-3 AUC: 0.8022	Ctrl
> 6.466 pg/μg TIMP-1 AUC: 0.7790		< 0.755 pg/μg MMP-13 AUC: 0.7668	< 0.417 pg/μg MMP-13 AUC: 0.7882	> 0.002 pg/μg HB-EGF AUC: 0.7105	< 0.019 pg/μg TIMP-4 AUC: 0.8750	JPA
> 265.2 pg/μg EGF AUC: 0.7791	> 0.755 pg/μg MMP-13 AUC: 0.7668		< 0.4163 pg/μg Angiopoietin-1 AUC: 0.7786	< 0.035 pg/μg HB-EGF AUC: 0.7143	< 0.019 pg/μg TIMP-4 AUC: 0.8750	MB
> 0.197 pg/μg MMP-3 AUC: 0.8900	> 0.417 pg/μg MMP-13 AUC: 0.7882	> 0.4163 pg/μg Angiopoietin-1 AUC: 0.7786		> 0.039 pg/μg HB-EGF AUC: 0.8636	> 0.039 pg/μg HB-EGF AUC: 0.8636	MM
> 215.2 pg/μg EGF AUC: 0.8213	< 0.002 pg/μg HB-EGF AUC: 0.7105	> 0.035 pg/μg HB-EGF AUC: 0.7143	< 0.039 pg/μg HB-EGF AUC 0.8636		< 0.019 pg/μg TIMP-4 AUC: 0.8750	AVM
> 0.321 pg/μg MMP-3 AUC: 0.8022	> 0.019 pg/μg TIMP-4 AUC: 0.8750	> 0.019 pg/μg TIMP-4 AUC: 0.8750	< 0.039 pg/μg HB-EGF AUC: 0.8636	> 0.019 pg/μg TIMP-4 AUC: 0.8750		DIPG

The table uses cut-off values (greater than or less than values) of various biomarkers to differentiate, and predict a disease given in the top row from those given in the right column. The intersecting cell shows the biomarker of significance, and AUC values depict strength of the diagnosis.

### Urinary biomarker species correlate with biological disparities between diseases

As can be seen in Table 2, discriminatory biomarkers between populations varied by cohort. These data reflected differences in the biological processes associated with specific diseases, including tissue remodeling, hypoxic response, and angiogenesis. Importantly, some of these processes are shared by different diseases at distinct times during their pathophysiologic progression. Consequently, we stratified our disease panel by the time course of the clinical progression of each disease, ranging from indolent (progression over > 6 months to years) to aggressive (progression within weeks to < 6 months). We then identified the biomarkers found to be significantly different between our control population and each disease in our study.

**Figure 2:**
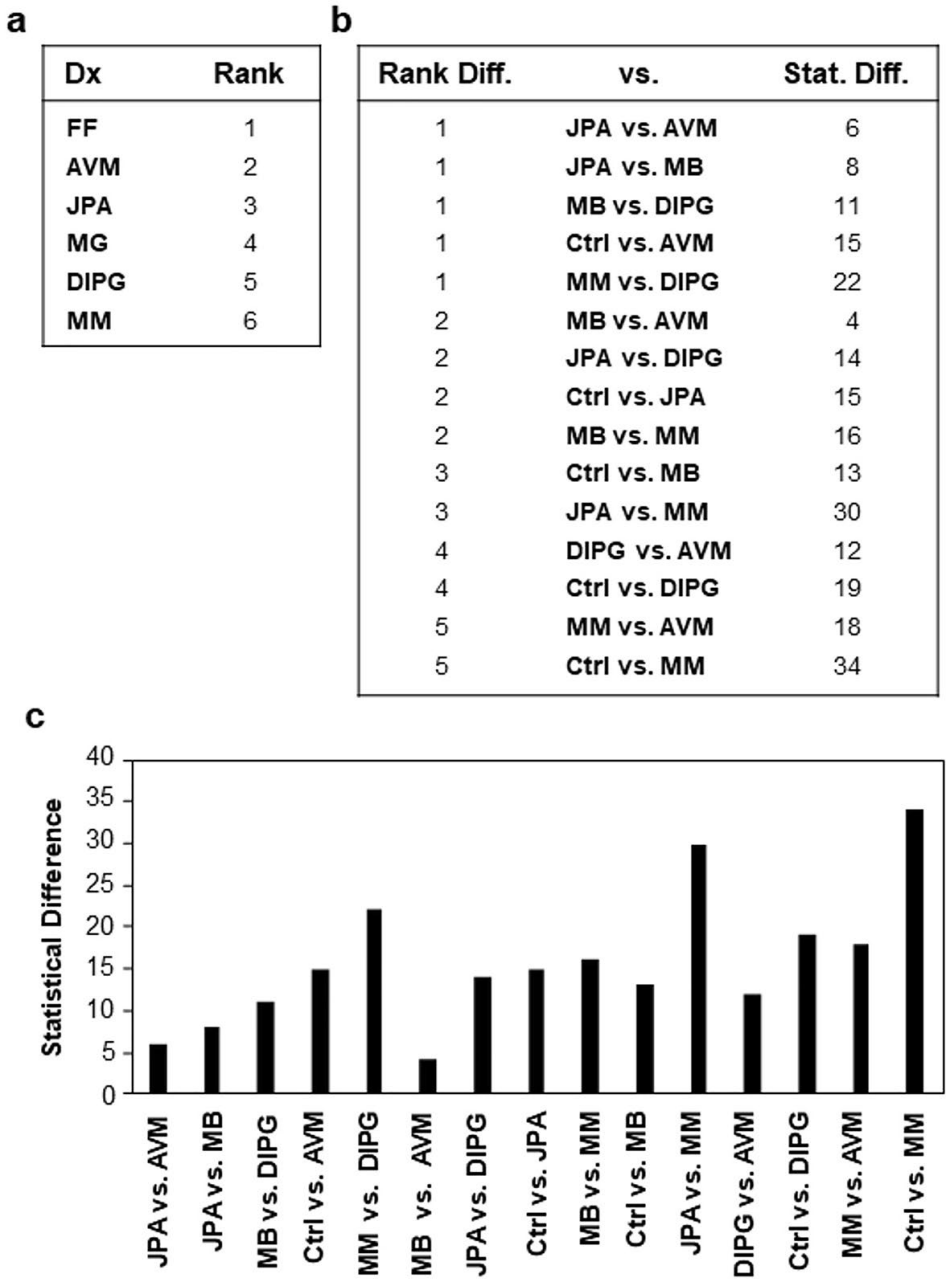
(**A**) Ranking of the diseases included in the study according to their relative indolence or aggression, as measured by time course of the disease. (**B**) Table ordering the comparisons in our study by the difference between the diseases, or the difference between disease and control, as measured by the absolute value of the difference between their ranking numbers. (**C**) Graph showing the statistical differences of each comparison, as measured by summing the statistical significance of the biomarkers which differentiate the two conditions being compared. This was done using the standard statistical notation of asterisks, where *p < 0.05, **p < 0.01, ***p < 0.001, and ****p < 0.0001, and summing the number of total asterisks per comparison, reflecting the amount of biological difference between indolent and aggressive disease.

Figure 2 highlights temporal progression of disease subtypes in biomarker panels. Diseases with rapid temporal progression demonstrate a greater number of putative biomarkers, reflecting the dynamic nature of multiple related processes (angiogenesis, invasion, growth, etc.) requiring elaboration of proteins seminal to several mechanistic pathways at the same time. These data highlight that urinary biomarkers accurately reflect the relative biological activity of a given disease and present a representative “snapshot” of the molecular milieu at the time of sampling.

## Discussion

### Background and purpose of the research

**Figure 3:**
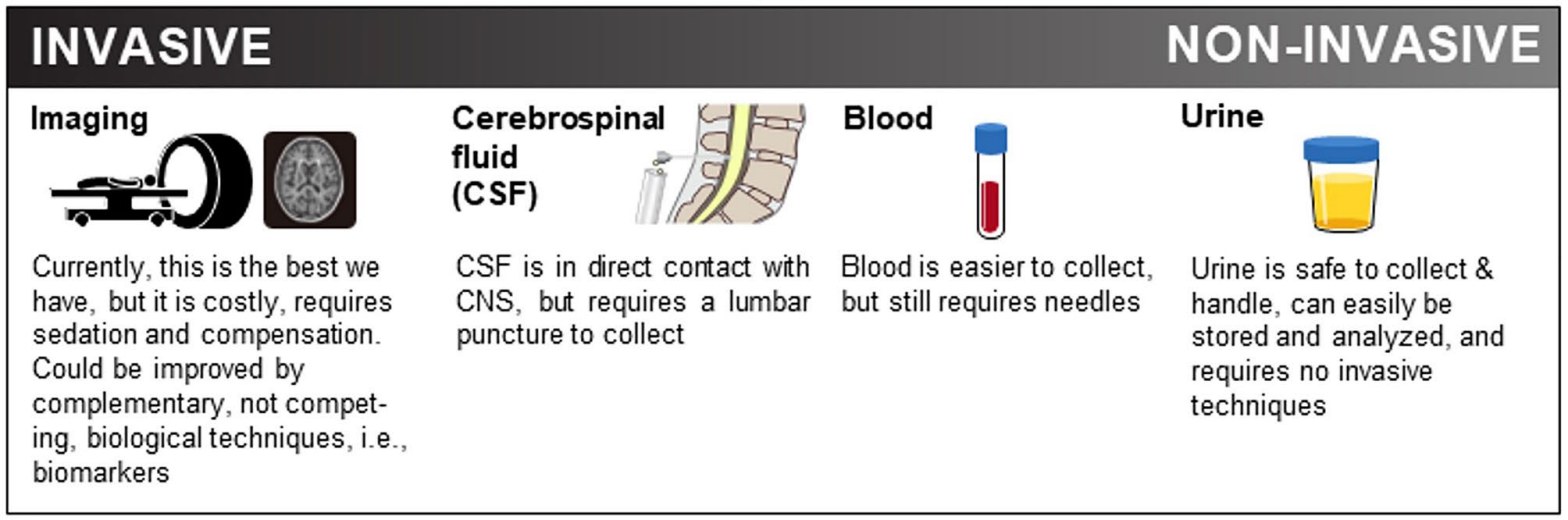
Reasons for using urine for pediatric brain tumors diagnostic.

There is a profound clinical need for the development of better methods to assist in the diagnosis, prognosis and treatment of diseases, particularly in the central nervous system (CNS). Diseases of the CNS persist as major sources of morbidity and mortality (such as brain tumors, which are the leading cause of non-trauma/poisoning related death in children)^12^. In addition, the CNS remains relatively inaccessible compared to other organ systems for direct examination, outside of expensive imaging or invasive sampling such as lumbar puncture or surgical biopsy. These challenges have spurred research into novel approaches, such as non-invasive biomarkers, to complement and improve upon current clinical practices (Fig. 3).

Our laboratory has had a longstanding interest in the development of non-invasive biomarkers designed to aid in the diagnosis, prognosis and therapy of tumors and cerebrovascular disease, including biomarker “fingerprints” that can distinguish between central nervous system tumors, moyamoya disease and arteriovenous malformations, including the first report of successfully applying this novel methodology specifically to brain tumors in a multicenter trial^1–3,5,6,13–19^. These previous studies have included tissue validation and longitudinal studies, showing changes in biomarker levels over time in direct correlation with changes in clinical status. However, despite these initial successes, a knowledge gap that remains centers on the specificity of these biomarkers across disease cohorts. Put another way, we have been able to look in detail—with tissue validation and longitudinal studies—deeply at individual conditions, but we have not compared broadly across different conditions.

Thus, there is a need to ascertain whether urinary biomarkers can distinguish between different conditions—and, ideally, to also discriminate subtypes within a condition (such as discerning between different tumor pathologies). The ability to provide disease-specific biomarker “fingerprints” would provide value in improving diagnostic accuracy and could also serve to identify potential therapeutic targets. Consequently, this project was undertaken to provide further proof-of-principle of the utility of urinary biomarkers, with a particular focus on demonstrating their ability to discriminate specific diseases, spanning both related and disparate conditions of clinical importance.

### Rationale for the use of urinary biomarkers

**Figure 4:**
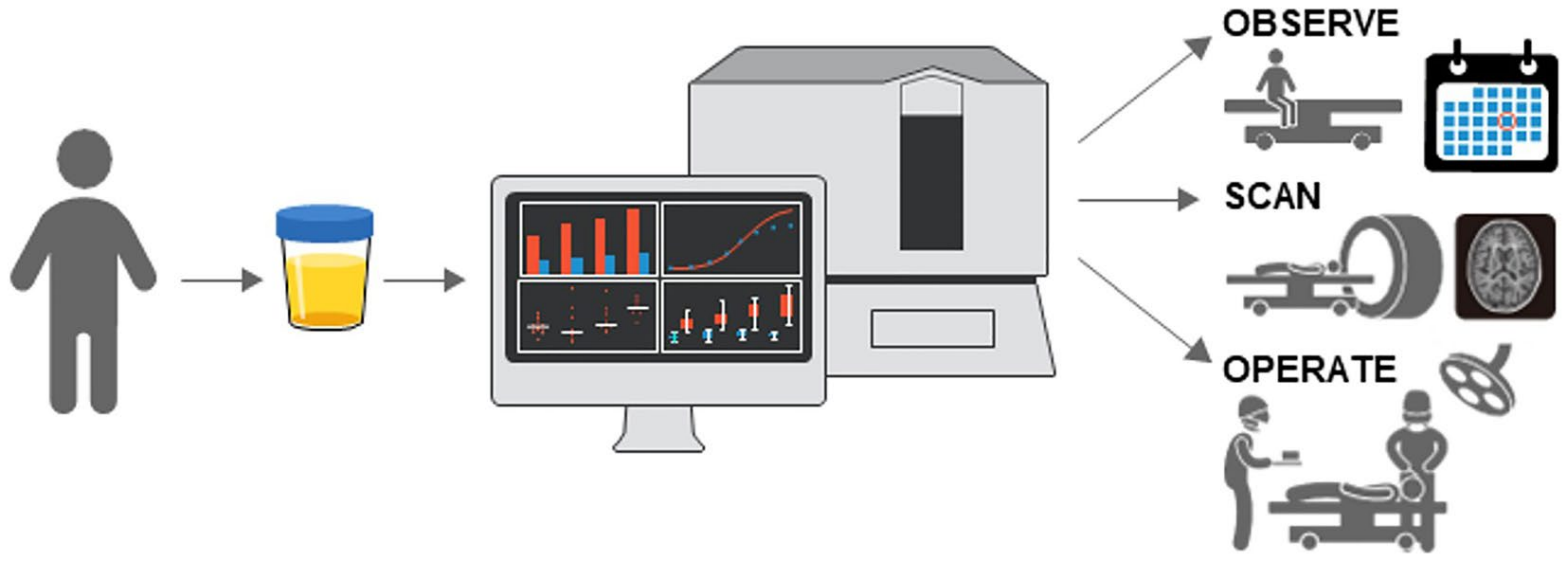
Schematic illustration for urinary biomarkers and their potential for the non-invasive detection of diseases.

Important to our research is the decision to use urine as the sampling medium. There is ample precedence for the successful use of urinary biomarkers to identify physiologic states such as pregnancy and to monitor disease, such as diabetes^20–23^. Use of urinary biomarkers for neurological disorders, particularly in pediatrics, offers a novel approach and provides advantages particularly relevant to this population. Current methods of brain tumor diagnosis and follow-up center around the use of infrequent clinical examinations and expensive radiographic studies, such as computerized tomography (CT) and magnetic resonance imaging (MRI) that often require sedation or anesthesia in children. By contrast, urine collection carries no risks to the patient and is far less expensive (at our institution, urine collection and analysis costs approximately 100-times less than MRIs) so it can easily be done at shorter intervals than are currently practical for imaging studies, potentially enabling earlier detection of recurrent disease and dynamic evaluation of current response to therapy. Collection of urine specimens is easy and non-invasive, avoiding the difficulties and risk inherent to lumbar punctures and blood tests. Urine collection can be done locally and mailed, saving families travel to tertiary care centers. Analysis is fast, (< 24 h), and results can be posted electronically without large or complex data sets (such as exist with imaging). Test results are numerical and are compared to specific statistical cutpoints, obviating the need for analysis that relies on subjective measures, such as film review or tissue staining. Significantly, biomarkers provide a method of assessment that relies on metabolic activity; a different—and complementary—approach to the current method of visual evaluation made with imaging studies (Fig. 4).

### Urinary biomarker panel selection rationale

The putative biomarker panel in this study was strategically selected based on known excretion in the urine, evidence that they are associated with the diseases under investigation and availability of commercially validated assays. The 21 molecules are all detectable in the urine, as demonstrated by our lab and others. All have been shown to play roles in the origin and pathology of neoplastic and vascular diseases (Table 5). It is worth discussing that an alternative approach includes indiscriminate screening of all urinary proteins to more broadly capture potential markers. We recognize the value of this approach and utilize it in our research. However, given the direct clinical focus of this project, we sought to ensure that the putative biomarkers have commercially available assays, an important consideration in potential use for clinical studies and in facilitating more rapid external validation by other groups.

**Table 5:** Biomarkers tested in the current study and their reported roles in diseases.

Class of biomarker	Molecule	Role in disease
Matrix metalloproteases (MMPs)	MMP-2	Implicated in renal carcinoma^24^, brain cancer^6^, tumor growth and progression, extracellular matrix remodeling^7,25^, and vascular anomalies^26^
	MMP-3	Implicated in cancer cell motility and invasion^27^, and tumor growth progression^25^
	MMP-9	Implicated in renal carcinoma^24^, brain cancer^6^, tumor growth and progression through extracellular matrix remodeling^7,25^, and vascular anomalies^26^
	MMP-13	Implicated in tumor metastasis and tumor growth and progression^25,28^
Growth factors, chemokines, and cytokines	Angiogenin	Implicated in cell proliferation and angiogenesis in cancer cells^29,30^
	Angiopoietin-1	Implicated in tumor angiogenesis and outcomes in cases of cerebral ischemia^31,32^
	EGF	Implicated in the stimulation of tumor angiogenesis through VEGF^33^
	bFGF	Implicated in angiogenesis and as a biomarker of a variety of cancers^26,34,35^
	HB-EGF	Implicated in the invasion, proliferation, and motility of cancer cells via the EGF pathway^36,37^
	HGF	Implicated in lymphangiogenesis^38^, metastasis^38^, invasion^39^, and epithelial-to-mesenchymal transition in cancer^40^ via the MET oncogene
	Netrin-1	Implicated as an upstream regulator of a tumor cell invasion and migration, and a biomarker for brain cancer^3^
	PIGF	Implicated in tumor angiogenesis^41^, and has been detected as a circulating biomarker for brain cancers^42^
	Prosaposin	Implicated in inhibition of tumor metastasis, and as an elevated serum protein in cancer patients^43–45^
	Thrombospondin-1	Has a complicated role in cancer, being shown by some to inhibit metastasis, progression, and angiogenesis^43,46–49^, and by others to increase invasion^50^. In either case, it has been shown to serve as an informative biomarker for cancer^51^
	Thrombospondin-2	Implicated in the downregulation of invasion and angiogenesis^48,52–54^, and accepted as a biomarker of cancer^55,56^
VEGF	A potent angiogenic regulator in tumors^57,58^, and also a known biomarker for a variety of cancers, including brain lesions^6,26,34^	
Cysteine proteases	Cathepsin B	An enzyme implicated in the invasiveness of glial-derived tumor cells through matric remodeling^59^
Tissue inhibitors of metalloproteases (TIMPs)	TIMP-1	Implicated in invasiveness and metastasis of cancer cells, and as markers of high grade cancers^24,60^
	TIMP-2	Implicated as a marker for carcinoma^24^
	TIMP-3	Implicated in the suppression of tumor growth and metastasis^61–63^
	TIMP-4	Implicated in stimulation of tumor growth^64^

### Potential applications to clinical practice

The study performed here provides several key findings that have relevance to clinical practice. First, while far from comprehensive, the disparate array of diseases included here represent the largest spectrum of CNS disorders in a single study of urinary biomarkers to date, more robustly validating the efficacy of this approach and better simulating the variety of conditions encountered in clinical practice. This supports the further investigation of urinary biomarkers in clinical trials (as has been done successfully for DIPG^13,65^) by underscoring the potential to develop disease-specific “fingerprints” and the applicability to a broad range of pathophysiological processes.

Second, these data indicate that there is the potential for creating screening tools that leverage the specificity identified in this work. While general population-based screening for relatively rare diseases such as these may not be currently practical, this work suggests that there may be the option to develop biomarker panels to target specific high-risk populations. A particularly relevant example are families at risk of developing moyamoya. While current genetic screening (such as looking for mutations in RNF213) can identify at-risk individuals, it cannot distinguish between non-affected carriers and those with active disease^66^. The addition of urinary biomarker testing might provide the ability to reveal which of these at-risk patients may have currently active disease, offering an option that is cheaper and easier to perform than MRI. Given that millions of individuals harbor this mutation (particularly those of Japanese, Chinese and Korean ancestry), there could be great value in developing this non-invasive tool. Another example of utility is illustrated with DIPG. As demonstrated in our previous study—and further validated here—the presence of DIPG can be detected and tracked with urinary testing^13,65^. This is especially important for children with this tumor, as using MRI studies for tumor burden when the patients require sedation and intubation is far riskier when they have brainstem disease that affects airway function. Using urinary biomarkers as an adjunct for tracking disease could potentially reduce the frequency of MRI studies, saving both cost and risk in this population.

Ultimately, we would anticipate a panel of biomarkers for a given disease, with distinct combinations employed based on the clinical need. For example, one fingerprint might be applied for screening, while a different group of biomarkers might help to stratify risk and follow response to surgery or treatment, as evidenced by previous clinical trial data^13,65^.

Finally, the role of urinary biomarkers may extend beyond diagnostic or prognostic adjuncts and actually inform the development of novel, biologically-based therapies. This approach of combining a specific therapy with immediate feedback on efficacy—theranostics—has rapidly expanded in medicine. Our lab has started to merge the fields of diagnostic biomarkers with targeted therapeutics in brain cancer^2,3,5,6,17–19,67^. It is tempting to consider that a similar approach with other CNS disorders, such as using biomarker-informed delivery of pro-angiogenic therapeutics in moyamoya or treating tumors with targeted chemotherapeutic agents, then following response to therapy using non-invasive urinary biomarkers as a complement to current imaging methods.

### Limitations and future directions

The data generated from this research is promising, but there are some limitations inherent to this work. First is the inherent rarity of the diseases. While they are of immense clinical impact, the relatively small cohorts limit generalizability of the conclusions. Future work should build on this encouraging preliminary data to drive multicenter collaborations to increase the number of patients for validation. Second is the limitation of the size of the panel of molecules to be assessed as putative biomarkers. The equipoise between the benefit of expanding the number of molecules to find new candidate biomarkers and the risk of an overwhelming volume of analysis to perform (or the addition of molecules with less robust assays for reliable detection) remains an ongoing challenge. We are currently approaching this challenge through the application of high-throughput screening technologies, which are continually evolving. Third is the question of how these biomarkers may change with time, and whether changes in levels—if they occur—reflect changes in disease status. We have data from select conditions (such as moyamoya, AVMs and brain tumors^1–4,13,17,65^ indicating that these biomarkers do accurately track response to therapy, but validation across larger populations and more conditions will be important.

To be clear, we are not advocating that this methodology would replace current practice, such as imaging studies or biopsy (when needed). Rather, our hope is that this approach would serve as a complement to these modalities, increasing accuracy and decreasing cost—ideally for the benefit of patients and clinicians alike.

## Conclusions

We report a novel panel of urinary biomarkers that can distinguish between a range of common, clinically relevant CNS diseases with high sensitivity and specificity. These data demonstrate proof-of-principle evidence that disease-specific urinary biomarker signatures exist. This work has the potential to enhance the diagnostic, prognostic and therapeutic capabilities of clinicians though the use of disease-specific biomarker panels, which can be assessed non-invasively, offering unique advantages in safety, ease of monitoring and reduced cost, along with a new quantifiable, biological approach that complements existing clinical and radiographic practice.

### Supplementary Information

Supplementary Information.

## Data Availability

All data generated or analyzed during this study are included in this published article and its supplementary information files S1.
