# Supplementary material for: Disease specific urinary biomarkers in the central nervous system
Source: Sci Rep. 2023 Nov 7;13:19244. doi: 10.1038/s41598-023-46763-z (PMC10630515; doi:10.1038/s41598-023-46763-z)
Supplement: Supplementary file 1 — Supplementary Information. [file 41598_2023_46763_MOESM1_ESM.pdf]

Supplemental Figure 1

a

Dipstick Legend

Abbrev.	Analyte	PLOCC*
A(1)	Angiopoietin-1	0.4163
A(2)	Angiopoietin-1	2.207
E	EGF	366.3
F(1)	FGFb	0.8443
F(2)	FGFb	1.074
H(1)	HGF	0.032
H(2)	HGF	0.05
M(1)	MMP-13	0.0625
M(2)	MMP-13	0.2347
M(3)	MMP-13	0.4038
N(1)	Netrin-1	0.1616
N(2)	Netrin-1	0.219
N(3)	Netrin-1	0.2601
T(1)	TIMP-3	1.998
T(2)	TIMP-3	2.079

******Protein Level of Color Change*

b

Representative example of a dipstick from a control (FF) patient

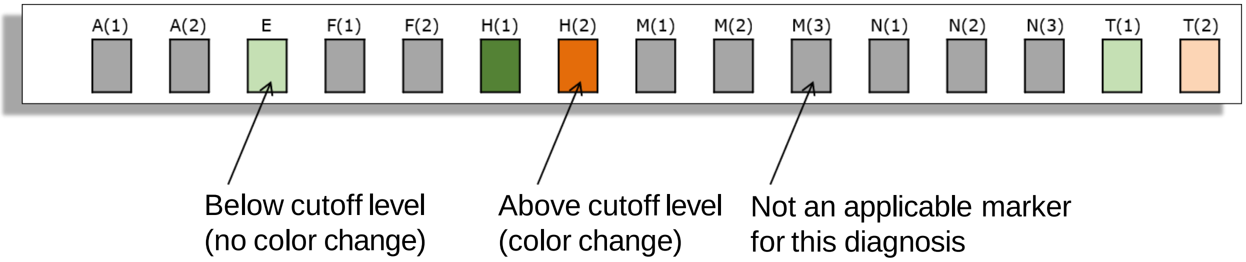

Supplemental. Figure 1. Possible translation of statistics into a urinary dipstick format.

Supplemental Figure 2

JPA
MB
MM
AVM
DIPG

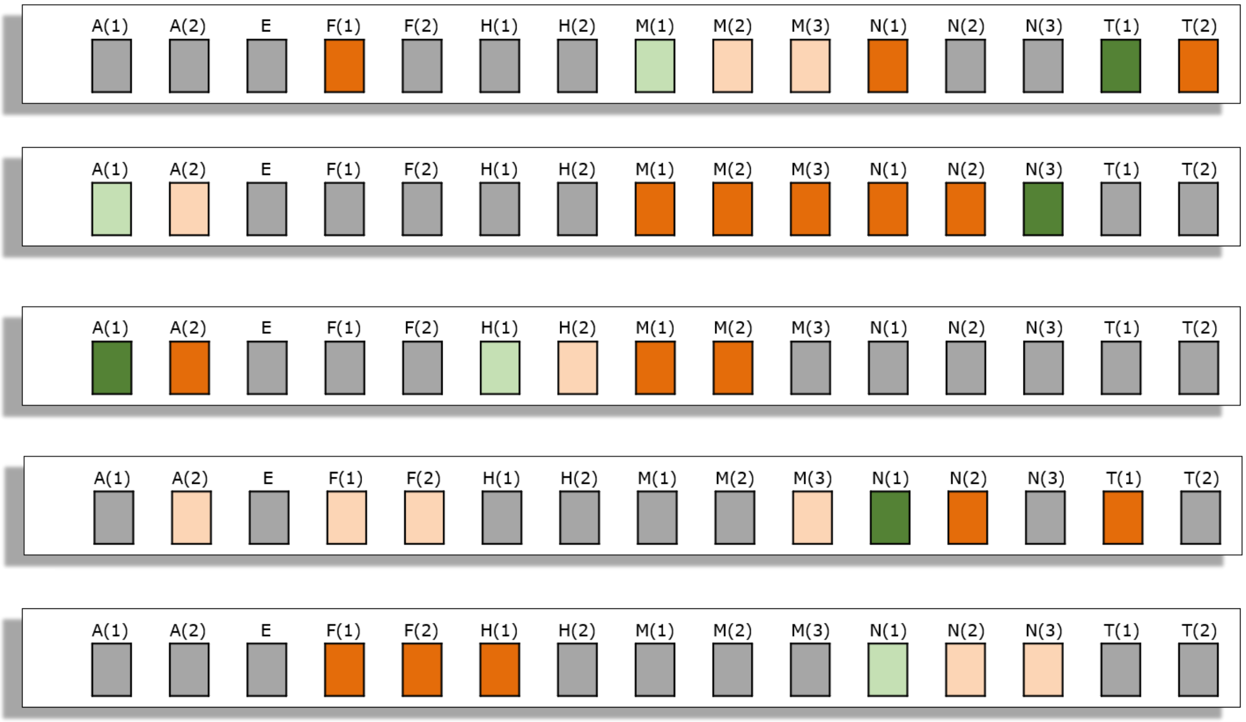

Supplemental. Figure 2. Representative examples of dipsticks from diseased patients graphically illustrating how the fingerprints for various diseases are distinct.

Supplemental Table I.

	Ctrl vs. JPA	Ctrl vs. MB	Ctrl vs. MM	Ctrl vs. AVM	Ctrl vs. DIPG	JPA vs. MB	JPA vs. MM	JPA vs. AVM	JPA vs. DIPG	MB vs. MM	MB vs. AVM	MB vs. DIPG	MM vs. AVM	MM vs. DIPG	AVM vs. DIPG
**Angiopoietin -1**			***			*	**			****			***	**	
**PIGF**			*		*										
**MMP-2**															
**MMP-3**		*	****		*		**						**		
**MMP-9**			**		*		***	*	**						
**MMP-13**		**	**			**	****				*	**	**	***	
**TIMP-1**	***	**			***		****			***			*	****	*
**TIMP-2**															
**TIMP-3**							*							*	
**TIMP-4**					**				**			**		***	***
**HGF**															
**VEGF**														**	**
**Netrin-1**					*				***			*		**	***
**EGF**															
**bFGF**	**		**		*										
**Angiogenin**															
**Angiopoietin-2**			**	*										*	
**Thrombospondin-1**		*	****	*			***							**	
**Thrombospondin-2**			***				**			*					
**HB-EGF**			****				***		*	*		*	**	****	

Supplemental Table I. Heat map showing the proteins which are significantly different comparing two diseases or a disease to control before to trim the data. *p<0.05 = *, p<0.01 = **, p<.001 = ***, and p<.0001 = *****
